# Big Five personality predict epilepsy diagnosis in 7  years

**DOI:** 10.3389/fneur.2023.1083792

**Published:** 2023-06-12

**Authors:** Weixi Kang

**Affiliations:** Department of Brain Sciences, Imperial College London, London, United Kingdom

**Keywords:** Big Five, personality, neuroticism, epilepsy, risk factor

## Abstract

**Objective:**

Recently, there is growing interest in investigating how personality traits could predict a subsequent diagnosis of various diseases. Regarding epilepsy, there is only preliminary evidence based on cross-sectional studies linking personality traits to epilepsy, hence, emphasizing the need for longitudinal studies. The aim of the current study is to assess if the Big Five personality traits can predict the risk of an epilepsy diagnosis.

**Methods:**

The current study analyzed data from 17,789 participants who participated in Understanding Society: the UK Household Longitudinal Study (UKHLS) at Wave 3 (collected between 2011 and 2012) and Wave 10 (collected between 2018 and 2019). The mean age was 47.01 (SD = 16.31) years and were 42.62% male. Two binary logistic regressions were used by including age, monthly income, highest educational qualification, legal marital status, residence, and standardized personality traits scores at Wave 3 as predictors for a clinical diagnosis of epilepsy at Wave 10 for males and females, respectively.

**Results:**

There were 175 participants (0.98%) with epilepsy and 17,614 participants (99.02%) without epilepsy at Wave 10. Results of the binary regression analyses revealed that Neuroticism is positively related to the risk of an epilepsy diagnosis in males (OR = 1.32, *p* = 0.04, 95% CI [1.01, 1.71]) but not in females 7  years after Wave 3 at Wave 10. However, other personality traits including Agreeableness, Openness, Conscientiousness, and Extraversion were not significant predictors of epilepsy diagnosis.

**Conclusion:**

These findings suggested that personality traits might enhance our understanding of psychophysiological associations in epilepsy. Neuroticism might be a relevant factor that should be taken into account in epilepsy education and treatment. Moreover, sex differences must be taken into account.

## 1. Introduction

Personality traits capture the basic individual differences in terms of the way people perceive, feel, think, and behave. The widely used Big Five model of personality consists of five broad traits/domains including Neuroticism (i.e., the tendency of being emotionally unstable), Agreeableness (the tendency to be cooperative, altruistic, and empathic), Openness (i.e., the tendency to taking novel routes), Conscientiousness (the tendency of being task-oriented and organized), and Extraversion (i.e., the tendency of being assertive and sociable) (1, 2). As one of the most widely used personality models in the literature, the Big Five can be also translated into other personality models as well (2–4). Recently, there are growing interest in investigating how personality traits could predict a subsequent diagnosis of various diseases. For instance, it has been suggested that Big Five personality traits can predict subsequent diseases later in life such as having a stroke, a heart condition, and a lung disease (5).

Personality traits can also be risks for an epilepsy diagnosis. Indeed, there are some studies that have looked at the associations between epilepsy and personality traits and found that there are associations between epilepsy and personality traits (6–12). Specifically, studies tend to find that people with epilepsy have higher levels of Neuroticism (6–12) and aggression (9) compared to healthy controls. In addition, a few studies have also found people with epilepsy are characterized by lower Openness compared to people without epilepsy (7, 10) and people with non-epilepsy seizures (12).

Thus, although there are some studies that compared the differences in personality traits between people with and without epilepsy, much less is known about how personality traits could prospectively predict the risks of epilepsy diagnosis in 7 years given the associations between epilepsy and personality traits can be bi-directional. Moreover, sex differences must be considered as women tend to have higher Extraversion, Agreeableness, and Neuroticism scores than men (e.g., (13)). Moreover, women have a slightly lower prevalence of epilepsy than men (e.g., (14)). The aim of the current study is to assess how Big Five personality traits could predict a diagnosis of epilepsy in 7 years.

## 2. Methods

### 2.1. Data

This study used data from Understanding Society: the UK Household Longitudinal Study (UKHLS), which has been collecting annual information from the original sample of UK households since 1991 (15). Participants completed the demographics and personality questions at Wave 3, which was collected between 2011 and 2012, and questions regarding if they have been clinically diagnosed with epilepsy at Wave 10, which was collected between 2018 and 2019. All data collections have been approved by the University of Essex Ethical Committee. Participants completed informed consent before participation in all studies. This data set is publicly available at https://www.understandingsociety.ac.uk. Participants with any missing variables of interest were removed from further analysis. Thus, there were 17, 789 participants with a mean age of 47.01 (SD = 16.31) years old with 42.62% males remaining for further analysis.

### 2.2. Measures

#### 2.2.1. Personality traits

Personality was measured using the 15-item version of the Big Five Inventory (16) with a Likert scale ranging from 1 (“disagree strongly”) to 5 (“agree strongly”). Scores were reverse-coded when appropriate. The exact set of questions used to ask participants can be found at: https://www.understandingsociety.ac.uk/documentation/mainstage/dataset-documentation/term/personality-traits?search_api_views_fulltext=. Mean scores were used for each of these traits. All personality scores were standardized (mean = 0, SD = 1) before further analysis.

#### 2.2.2. Epilepsy

Participants answered the question “Has a doctor or other health professional ever told you that you have any of these conditions? Epilepsy.” Self-reported epilepsy is a valid measure to assess epilepsy status in cohort studies (17) with good positive predictive value, good sensitivity, and specificity.

#### 2.2.3. Control variables

Control variables in the model include age (continuous), sex (male = 1 vs. female = 2), monthly income (continuous), highest educational qualification (college = 1 or below college = 2), legal marital status (single = 1 vs. married = 2), residence (urban = 1 vs. rural = 2), and life satisfaction (on a scale from “1 = completely dissatisfied” to “7 = completely satisfied”).

### 2.3. Analysis

Personality traits including Neuroticism, Agreeableness, Openness, Conscientiousness, and Extraversion scores and life satisfaction were standardized before (mean = 0, SD = 1) further analysis. Two binary logistic regressions were used by taking demographics including age, monthly income, highest educational qualification, legal marital status, residence, and standardized personality traits scores at Wave 3 predictors to predict ever clinical diagnosis of epilepsy at Wave 10 for males and females, respectively.

## 3. Results

The mean age of these participants is 47.01 with a standard deviation of 16.31. The average monthly income of these participants is 1465.98 with a standard deviation of 1381.43. The mean life satisfaction score is 5.19 with a standard deviation of 1.47. The average of personality traits scores before standardization is 3.58 for Neuroticism, 5.64 for Agreeableness, 4.58 for Openness, 5.51 for Conscientiousness, 4.58 for Extraversion with a standard deviation of 1.43, 1.02, 1.28, 1.08, and 1.30, respectively. In addition, 7,582 (42.62%) of them are males with 10,207 females (57.38%). 11,751 (66.06%) participants have not received a college degree whereas 6,038 (33.94%) of them received at least a college degree. Out of 11,789 participants involved in the study, 7,846 participants (44.11%) were single whereas 9,943 (55.89%) were married. There were also 12,421 participants living in the cities whereas 4,368 participants lived in rural areas (24.55%). Most importantly, 17,614 participants (99.02%) indicated that they have not been clinically diagnosed with epilepsy in Wave 10 whereas 175 participants (0.98%) indicated that they have been clinically diagnosed with epilepsy in Wave 10 (Table 1). The current study found that age is whereas Neuroticism is related to a higher risk of epilepsy diagnosis in a 7-year period (OR = 1.32, *p* = 0.04, 95% CI [1.01, 1.71]) in males but not in females (Table 2).

**Table 1:** Descriptive statistics for control variables and personality traits.

	Mean	SD
Age	47.01	16.31
Monthly income	1465.98	1381.43
Life satisfaction	5.19	1.47
Neuroticism	3.58	1.43
Agreeableness	5.64	1.02
Openness	4.58	1.28
Conscientiousness	5.51	1.08
Extraversion	4.58	1.30
	*N*	%
**Sex**		
Male	7,582	42.62
Female	10,207	57.38
**Highest educational qualification**		
Below college	11,751	66.06
College	6,038	33.94
**Legal marital status**		
Single	7,846	44.11
Married	9,943	55.89
**Residence**		
Urban	13,421	74.45
Rural	4,368	24.55
**Epilepsy at Wave 10**		
Yes	17,614	99.02
No	175	0.98

**Table 2:** The binary logistic regression results with demographics and personality traits at Wave 3 as predictors and whether one has been clinically diagnosed with epilepsy at Wave 10 as the predicted variable for males and females, respectively.

	Male			Female		
	OR	*p*	95% CI	OR	*p*	95% CI
Age	1.00	0.89	[0.99, 1.02]	0.99	0.04	[0.98, 1.00]
Sex	1.00	0.25	[1.00, 1.00]	1.00	0.22	[1.00, 1.00]
Monthly income	0.94	0.82	[0.56, 1.59]	0.96	0.85	[0.60, 1.53]
Highest educational qualification	0.95	0.85	[0.58, 1.57]	0.87	0.54	[0.57, 1.34]
Residence	0.72	0.28	[0.40, 1.30]	1.21	0.41	[0.77, 1.91]
Life satisfaction	0.97	0.83	[0.75, 1.26]	0.97	0.76	[0.77, 1.21]
**Neuroticism**	**1.32**	**0.04**	**[1.01, 1.71]**	1.17	0.19	[0.93, 1.47]
Agreeableness	0.95	0.70	[0.73, 1.23]	1.10	0.42	[0.87, 1.41]
Openness	0.98	0.90	[0.76, 1.28]	0.93	0.55	[0.75, 1.17]
Conscientiousness	1.00	0.99	[0.76, 1.31]	0.85	0.18	[0.67, 1.08]
Extraversion	1.24	0.12	[0.95, 1.61]	1.05	0.65	[0.84, 1.32]

## 4. Discussion

Taken together, the aim of the current study was to assess whether personality traits serve as risk factors for epilepsy prospectively. By using a binary logistic regression to analyze data from 17,789 participants from UKHLS with a mean age of 47.01 (SD = 16.31) years old and 42.62% of males, the current study revealed that Neuroticism is positively related to the risks of epilepsy diagnosis in 7 years in males but not in females.

The findings from the current study are largely consistent with previous studies regarding the positive associations between epilepsy and Neuroticism (6–12) in both males and females. People who score high on Neuroticism tend to be emotionally unstable and are at great risk of developing depressive symptoms (18). These findings can be potentially explained by the association between mental health problems and epilepsy (e.g., (19, 20)). Moreover, Neuroticism is also associated with higher levels of inflammation (21), which then contributes to the risk of developing epilepsy in 7 years (See (22) a review). Moreover, Neuroticism is also associated with health-risk behaviors such as less physical activity and more sedentary behaviors (23), smoking (24, 25), and excessive alcohol use (26), which may then lead to a higher risk of epilepsy (27).

Despite some strengths of the current study including the use of a large, longitudinal sample that assessed clinical epilepsy diagnosis, there are some limitations that may motivate future research. First, measuring personality traits earlier in life over a longer period of time can further clarify the association between personality traits and epilepsy. Second, it is possible that epilepsy can occur and impact personality traits before the official diagnosis of epilepsy. Third, the current study focused on a sample from the United Kingdom, which may make it hard to generalize the current findings to other countries. Future studies should test if this association still exists in other countries. Finally, other risk factors of epilepsy including alcohol misuse, stoke, brain tumor, head injury were not controlled in the present study. Future studies should control these risk factors of epilepsy.

Taken together, the current study is among the first that has demonstrated that personality traits are predictors of subsequent clinical diagnosis of epilepsy. The current study found that Neuroticism is positively related to the risks of epilepsy diagnosis in 7 years in males but not in females. Thus, there is a need to include personality traits in routine assessments by health professionals, as they could identify individuals with a greater risk of developing epilepsy. Moreover, sex differences must be taken into account.

## Data availability statement

Publicly available datasets were analyzed in this study. This data can be found at: https://www.understandingsociety.ac.uk.

## Ethics statement

The studies involving human participants were reviewed and approved by University of Essex. Written informed consent to participate in this study was provided by the participants’ legal guardian/next of kin

## Author contributions

WK: conceptualization, data curation, formal analysis, investigation, methodology, resources, software, writing—original draft, and writing—review and editing.

## Funding

This work was supported by the Imperial Open Access Fund.

## Conflict of interest

The author declares that the research was conducted in the absence of any commercial or financial relationships that could be construed as a potential conflict of interest.

## Publisher’s note

All claims expressed in this article are solely those of the authors and do not necessarily represent those of their affiliated organizations, or those of the publisher, the editors and the reviewers. Any product that may be evaluated in this article, or claim that may be made by its manufacturer, is not guaranteed or endorsed by the publisher.
